# Supplementary material for: Direct and indirect effects of the COVID-19 pandemic on mortality in Switzerland
Source: Nat Commun. 2023 Jan 6;14:90. doi: 10.1038/s41467-022-35770-9 (PMC9817462; doi:10.1038/s41467-022-35770-9)
Supplement: Supplementary file 1 — Supplementary Information [file 41467_2022_35770_MOESM1_ESM.pdf]

Online Supplement: "Direct and indirect effects of the COVID-19 pandemic on mortality in Switzerland."

Julien Riou[1, 2], Anthony Hauser[1, 2], Anna Fesser[2], Christian L. Althaus[1], Matthias Egger[1, 3, 4], and Garyfallos Konstantinoudis [5,*]

[1] *Institute of Social and Preventive Medicine, University of Bern, Bern, Switzerland*

[2] *Federal Office of Public Health, Switzerland*

[3] *Population Health Sciences, Bristol Medical School, University of Bristol, Bristol, UK*

[4] *Centre for Infectious Disease Epidemiology and Research, University of Cape Town, Cape Town, South Africa*

[5] *MRC Centre for Environment and Health, Department of Epidemiology and Biostatistics, School of Public Health, Imperial College London, London, UK*

[*] Corresponding author. Email: g.konstantinoudis@imperial.ac.uk

Contents

S1 Text **3**

S1.1 Population models . . . 3

S1.1.1 Model specification . . . 3

S1.1.2 Cross validation . . . 3

S1.1.3 Results . . . 4

S1.2 Expected mortality model . . . 5

S1.2.1 Model specification . . . 5

S1.2.2 Prior specification . . . 6

S1.2.3 Cross validation . . . 6

S1.3 Sensitivity analysis . . . 10

S1.4 Separation model . . . 12

List of Tables

S1 Median and 95% credible intervals of β_1 and β_2 for the Total group, the different phases and age groups considered. . . . 13

S2 Median and 95% credible intervals of β_1 and β_2 and total number of observed all-cause deaths across the different cantons. . . . 14

S3 Number of deaths by age group and phase of the pandemic. . . . 15

List of Figures

S1 Association between weekly laboratory-confirmed COVID-19-related deaths and absolute excess mortality by phase. The black line shows the slope of association corresponding to a 1 to 1 relation. The red lines show the association estimated with the model (corresponding to the β_1 coefficients, the full line represents the point estimate and the dashed lines the lower and upper bounds of the 95% credible interval). . . . 16

S2 Mean and 95% credible intervals (error bars) of β_1 (panel A) and β_2 (panel B) across the different cantons. The canton abbreviations and sample size by canton are given in Table S2. n=156,193 number of observed all-cause deaths were used to derive the statistics. . . . 17

S1 Text

S1.1 Population models

S1.1.1 Model specification

Let P_{ijkl} be the population for the i-th sex (male-female), j-th age-group (<40, 40-59, 60-69, 70-79, $\geq$ 80), k-th year (2010-2019) and l-th canton. Let X_{1i} be the sex and X_{2k} be the year covariate. To predict populations for the years 2020-2023, had the COVID-19 pandemic not occurred, we considered 6 models of the following formulation:

$$P_{ijkl} \sim \text{Poisson}(\mu_{ijkl})$$

$$\log(\mu_{ijkl}) = \beta_0 + \beta_1 X_{1i} + \beta_2 X_{2k} + b_{jkl} \tag{1}$$

where b_{jkl} is a random effect that has an age, temporal and spatial structure, see Table 1. All random effects were assigned an iid structure: $w_j, v_k, u_l, \xi_{jkl}, \beta_{2jkl}, \sim N(0, \sigma_g^2), g = 1, \ldots, 5$ and for σ_g^2 we considered penalised complexity priors [1]. In particular, we selected a prior so that $\Pr(\sigma_t > 10) = 0.1$, implying that it is unlikely to have population counts larger than exp(10) based solely on the selected structure of the random effects. All the models were fit using the Integrated Nested Laplace Approximation (INLA) for quick computation [2].

Model	Abbreviation	b_{jkl}
1	BASE	$w_j + v_k + u_l$
2	OV	$w_j + v_k + u_l + \xi_{jkl}$
3	OV_INT	$w_j + v_k + u_l + w_j \otimes v_k + w_j \otimes u_l + v_k \otimes u_l + \xi_{jkl}$
4	VC	$w_j + v_k + u_l + \beta_{2jkl} X_{2k}$
5	VC_OV	$w_j + v_k + u_l + \xi_{jkl} + \beta_{2jkl} X_{2k}$
6	VC_OV_INT	$w_j + v_k + u_l + w_j \otimes v_k + w_j \otimes u_l + v_k \otimes u_l + \xi_{jkl} + \beta_{2jkl} X_{2k}$

Table 1. Specification of the random effects of the different population models. The notation $\otimes$ defines the Kronecker product for the interaction between two random variables.

S1.1.2 Cross validation

To mimic the scenario we want to reproduce, we selected to leave out the past 3 years (as we want to predict 3 years of population) all-together and examine the model prediction. To compare model prediction we calculated the coverage proportion, root mean square error (RMSE) and the mean bias. The coverage proportion is defined

as the probability that the true population value lies in the 95% model based credible intervals, the MSE as $E[(\tilde{Y}_{ijkl} - Y_{ijkl})^2]$ and the mean bias as $E[\tilde{Y}_{ijkl} - Y_{ijkl}]$, where $\tilde{Y}_{ijkl}$ are the predictions and Y_{ijkl} the true values of the population.

S1.1.3 Results

Table 2, shows the results of the cross validation and it is apparent that model 3, performs best, and this was the model selected for the subsequent analysis of excess deaths.

Model	Coverage	RMSE	mean bias
BASE	0.08	6198	15
OV	0.96	7481	145
OV_INT	0.98	6009	9
VC	0.38	7415	175
VC_OV	0.55	6265	17
VC_OV_INT	0.61	7694	139

Table 2. Coverage, root mean square error (RMSE) and mean bias for the different models based on the leave out the past 3 years cross validation scheme.

S1.2 Expected mortality model

S1.2.1 Model specification

We used a Bayesian hierarchical model to predict deaths in 2020-2022, under the counterfactual scenario of absence of the COVID-19 pandemic. Let Y_{jtkl} be the number of all-cause deaths, P_{jtkl} be the population at risk (for the weeks during 2010-2019 this is fixed, whereas we used 200 samples to propagate the model based uncertainty from the population model for the weeks during 2020-2022) and r_{jtkl} the risk in the j-th age-sex group (male-female and <40, 40-59, 60-69, 70-79, ≥ 80), t-th week ($t = 1, \dots, 595$, with 1 denoting the first week of 2011 and 595 the week that ends on the 3rd of April), the k-th year ($k = 1, \dots, 13$ with year 1 corresponding to 2011) and l-th canton ($s = 1, \dots, S$). We have to note that unlike the previous model, we did not group by sex in this analysis, as previous analysis showed small differences across the different sexes [3]. We assume a Poisson distribution for the number of deaths Y_{jtkl} and specified the following model:

$$Y_{jtkl} \sim \text{Poisson}\big(r_{jtkl} P_{jtkl}\big) \tag{2}$$

$$\log\left(r_{jtkl}\right) = \beta_{0t} + \beta_1 X_{1t} + \beta_2 X_{2j} + \beta_3 X_{3k} + f(x_{jtkl}) + v_t + u_l, \tag{3}$$

where β_{0t} is a week specific intercept given by $\beta_{0t} = \beta_0 + \epsilon_t$, with β_0 being the global intercept and $\epsilon_t \sim \text{Normal}(0, \sigma_\epsilon^2)$ an unstructured random effect representing the deviation of each week from the global intercept, with σ_ϵ^2 denoting the variance of ϵ_t. The term β_1 represents the effect of public holidays, β_2 the effect of age and β_3 a linear term to capture the long-term trend of the mortality. The effect of (mean weekly and cantonal) temperature on the all-cause mortality is captured in the flexible functions $f(\cdot)$. We define $f(\cdot)$ as a second-order random walk:

$$x_{tl} \mid x_{(t-1)l}, x_{(t-2)l}, \sigma_x^2 \sim \text{Normal}\left(2x_{(t-1)l} + x_{(t-2)l}, \sigma_x^2\right), \tag{4}$$

with σ_x^2 denoting the variance and x_{tkl} the temperature in the t-th week of the k-th spatial unit.

We accounted for seasonality using a non-linear weekly effect v_t with a first order random walk (RW1) structure:

$$v_t \mid v_{t-1}, \sigma_v^2 \sim \text{Normal}(v_{t-1}, \sigma_v^2), \tag{5}$$

where σ_v^2 is the variance of v_t.

The term u_l is defined as a reparametrisation of the Besag-York-Mollié model given by the sum of an unstructured random effect, $\gamma_l \sim \text{Normal}(0, \sigma_\gamma^2)$, and a spatially structured effect δ_l [4, 5]. In particular u_l is

defined as:

$$u_l = \sigma_u^2 \left(\sqrt{1-\theta}\gamma_l^\star + \sqrt{\theta}\delta_l^\star \right), \quad (6)$$

where $\gamma_l^\star$ and $\delta_l^\star$ are standardised version of γ_l and δ_l to have variance equal to 1 [1]. The term $0 \leq \theta \leq 1$ is a mixing parameter which measures the proportion of the marginal variance explained by the structured effect and σ_u^2 the variance of the spatial field.

S1.2.2 Prior specification

We specified uninformative priors for the fixed effects β_0, β_1 and β_2. For the hyperparameters of the random effects we considered priors that tend to regularise inference while not providing too strong information [1]. For the standard deviation of the spatial field we defined a prior so that $\Pr(\sigma_u > 1) = 0.01$, implying that it is unlikely to have a spatial relative risk higher than $\exp(2)$ based solely on spatial variation. For θ we set $\Pr(\theta < 0.5) = 0.5$ reflecting our lack of knowledge about which spatial component, the unstructured or structured, should dominate the field u. For the rest of the standard deviations we defined the priors as $\Pr(\sigma_* > 1) = 0.01$.

S1.2.3 Cross validation

To examine the predictive ability of the above-mentioned model we defined a cross validation scheme, leaving the past 2 years out (we did not leave a third year out, as our predictions for 2022 are only for the first 3 months). We used metrics regarding the coverage proportion, bias, and correlation between predicted and true value of deaths. The results of the cross validation are shown on Figures 1-5 and Table 3.

Figure 1 shows the correlation between predicted and true value of deaths by age, sex and year (across the weeks and cantons). We see that the correlation is highest for the older groups and is not modified by year or sex. As applying the Pearson correlation coefficient is less appropriate when counts are small, we also calculated the coverage proportion and bias. Figure 2 shows the bias by age, sex and year (across the weeks and cantons) and we see that it is always centred in 0. Figure 3 shows the coverage proportion by age, sex and year and we see that is pretty high for all groups, with the lowest (0.95) being for the older age groups. We also calculated the relative bias (truth-predicted / truth) by canton and and week and we can see that there the model is overall unbiased, Figure 4 and 5. Table 3 calculated the relative bias in the totals by age, sex in 2018, 2019 and 2018 and 2019 combined. We observed higher relative bias in females in general, but the overall relative bias for the totals is 0.

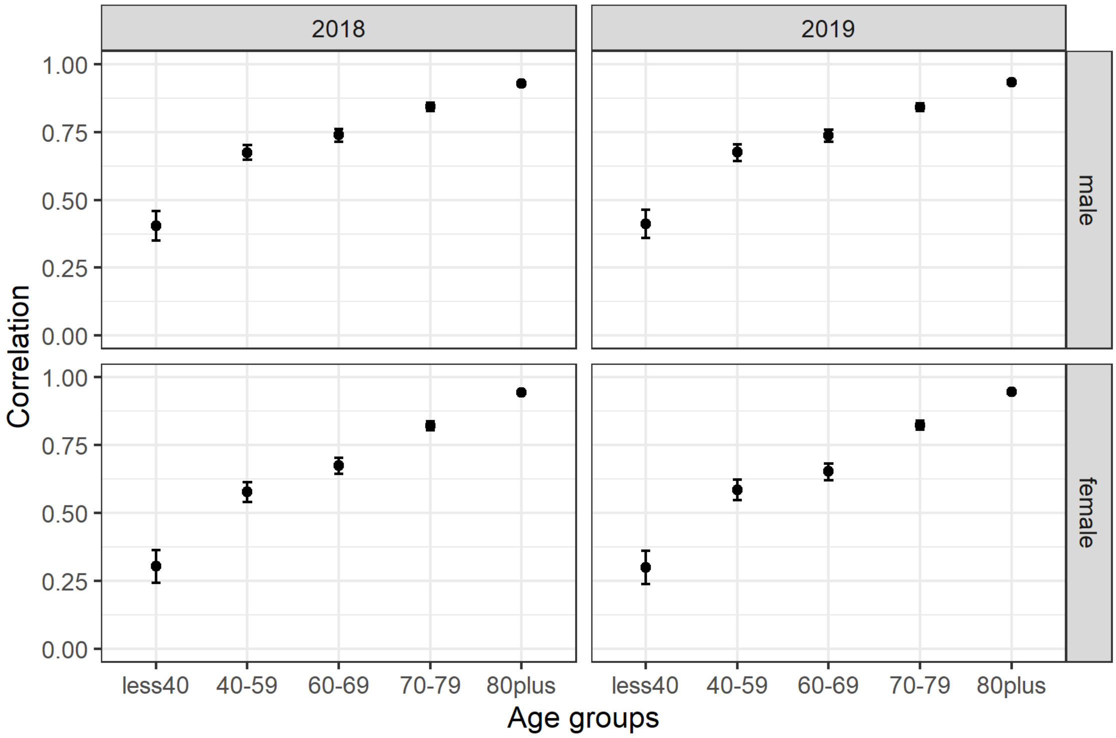

Figure 1. Correlation between the predicted and observed weekly and cantonal number of deaths by year, age and sex.

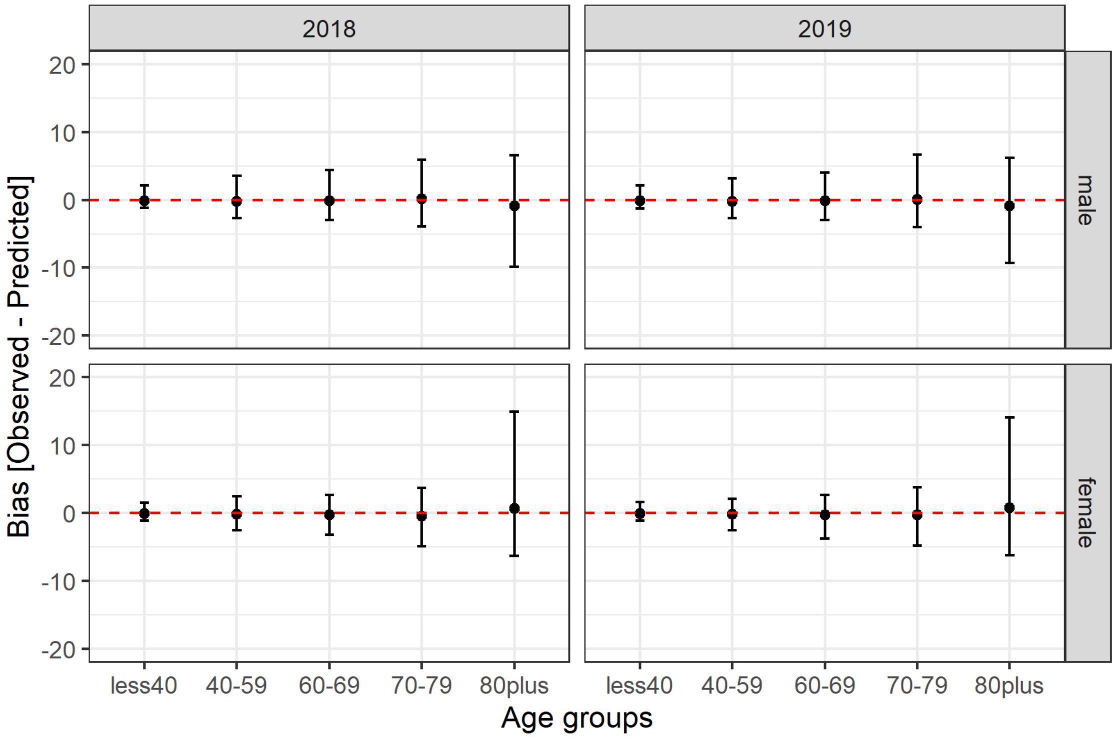

Figure 2. Bias between the predicted and observed weekly and cantonal number of deaths by year, age and sex.

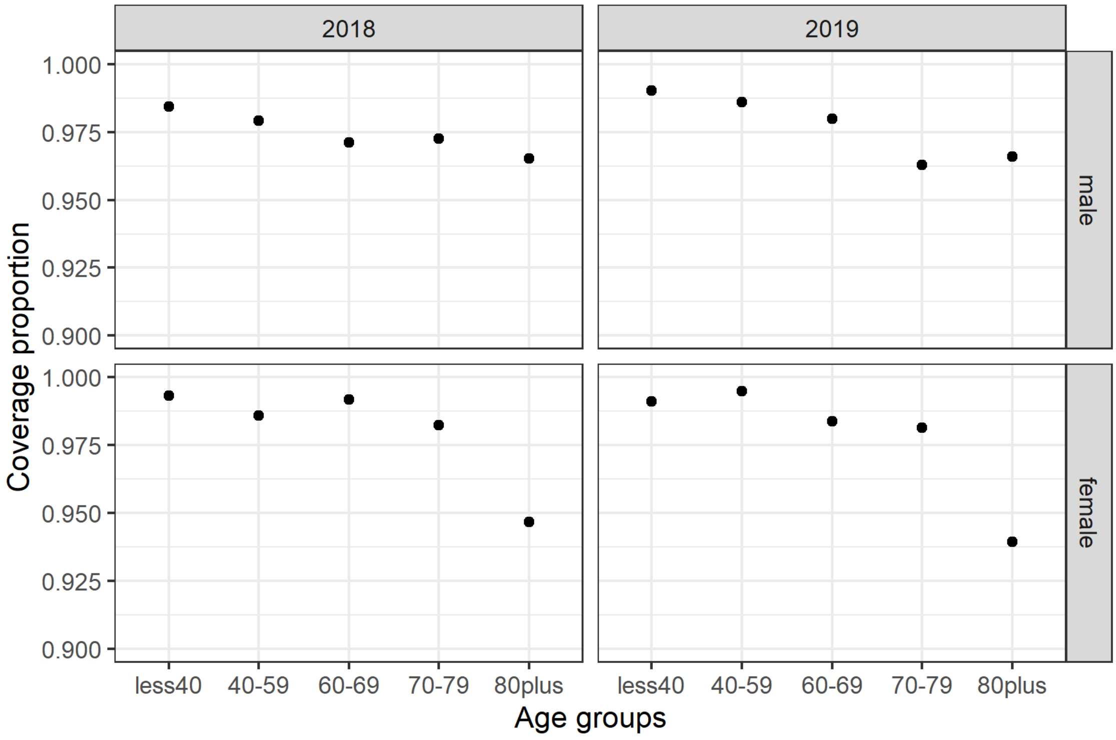

Figure 3. Coverage proportion by year, age and sex.

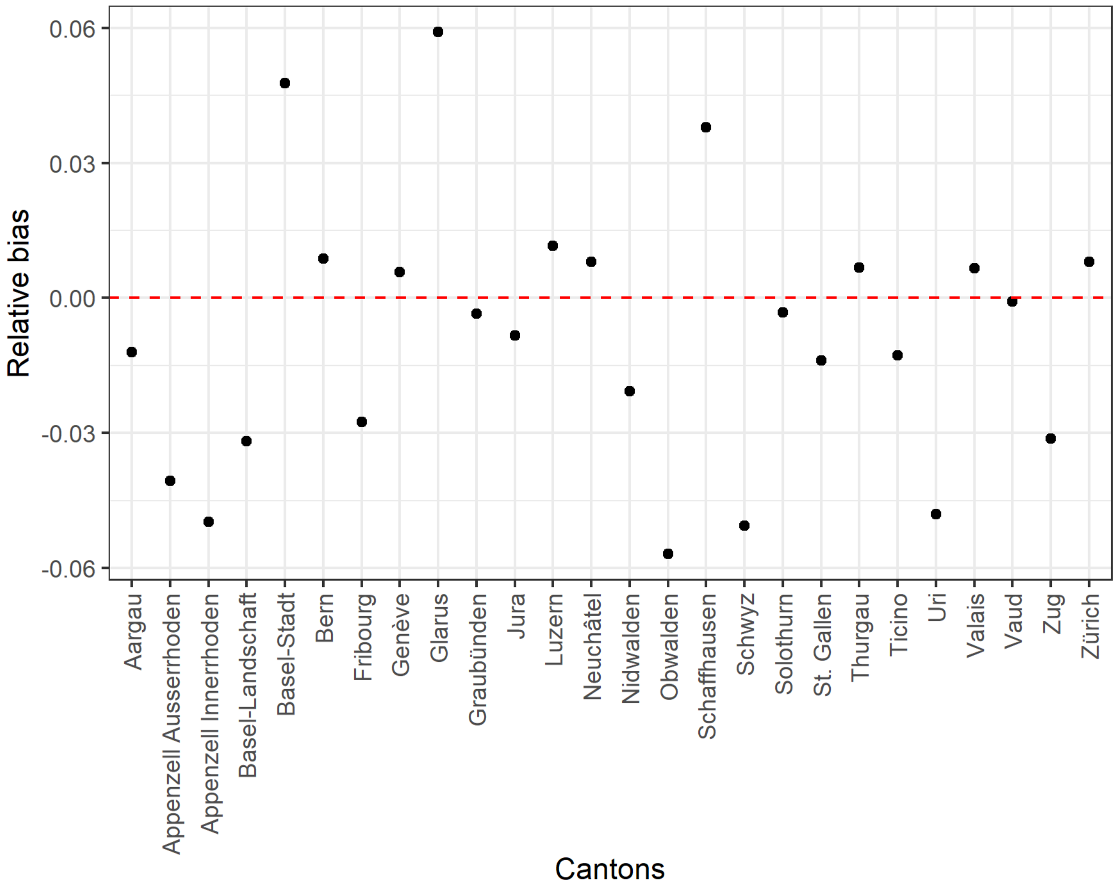

Figure 4. Relative bias by canton.

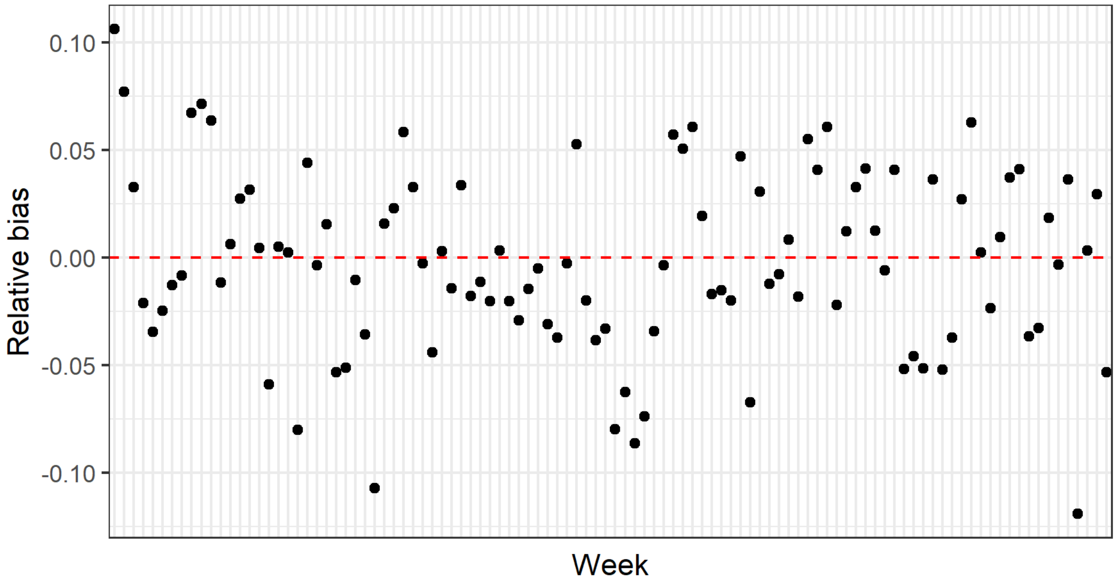

Figure 5. Relative bias by week.

age	sex	2018	2019	2018-2019
less40	male	0.05	0.01	0.04
40-59	male	0.04	0.00	0.02
60-69	male	0.07	0.04	0.05
70-79	male	0.06	0.07	0.07
80plus	male	-0.09	-0.10	-0.10
less40	female	-0.13	-0.22	-0.18
40-59	female	-0.11	-0.19	-0.15
60-69	female	-0.16	-0.19	-0.17
70-79	female	-0.11	-0.08	-0.10
80plus	female	0.08	0.09	0.08
less40	Total	-0.02	-0.07	-0.04
40-59	Total	-0.01	-0.07	-0.04
60-69	Total	-0.02	-0.05	-0.03
70-79	Total	-0.01	0.01	-0.00
80plus	Total	0.01	0.01	0.01
Total	Total	0.00	-0.00	-0.00

Table 3. Relative bias by age, sex and year.

S1.3 Sensitivity analysis

One limitation of the proposed approach is that it ignores the change in population caused by the excess deaths themselves. In that sense, an excess in mortality towards the beginning of the study period should decrease the population for the rest of the period, and failing to account for this decrease would result in an overestimation of the expected number of deaths towards the end of the study period, and thus an underestimation of excess mortality. This is especially important in groups that were heavily impacted in terms of mortality in the early period. We considered this issue in a sensitivity analysis where the population in 2020-2022 was updated each week by subtracting the cumulative excess deaths:

$$P^*_{j,l,t+1} = P_{j,l,t+1} - \sum_{u=1}^{t} \frac{E_{j,l,u}}{P_{j,l,u}} P^*_{j,l,u} \tag{7}$$

where $P_{j,l,t}$ is the population in age group j, week t (from the first week of 2020) and canton l estimated in section S1.1; E is the expected deaths estimated in section S1.2, and P^* is the population corrected for the cumulative excess deaths (we set $P^*_{j,l,t=0} = P_{j,l,t=0}$). With this approach, we correct population estimates over time, avoiding the overestimation of expected deaths and the underestimation of excess deaths.

The correction was only meaningful for age groups 70-79 and 80+ (Figure 6). Estimates of excess mortality were higher with this approach, with about 1,000 additional excess deaths (Table S3). As expected, the difference was more visible in the later phases (Table 4). This however had very little effect on the estimates of β_1 and β_2 (Table 5).

Figure 6. Population projections for 2020-2022 used in the model (red corresponds to the original approach explained in section 1.1, green corresponds to the correction for excess).

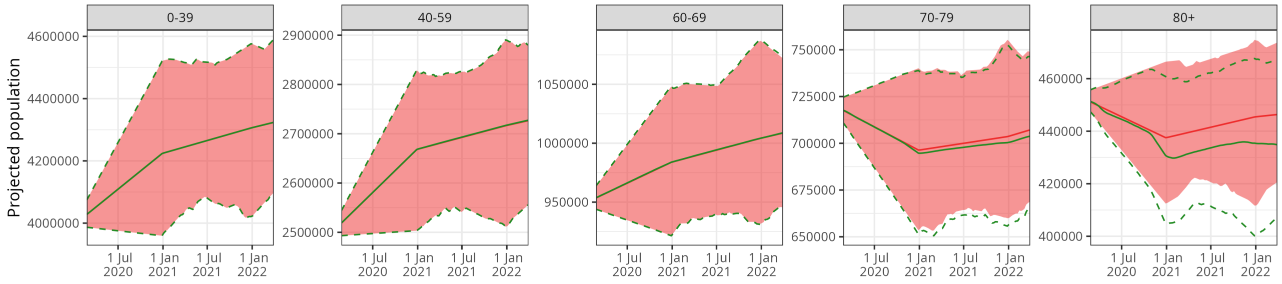

Table 4. Estimates of excess mortality obtained in the sensitivity analysis correcting population for excess.

Phase	Expected	Observed	Excess	Relative excess	Laboratory-confirmed COVID-19 deaths
1	19,368 (18,752 to 20,025)	20,791	1,423 (766 to 2,039)	7% (4 to 11)	1,725
2	19,164 (18,407 to 20,049)	19,103	-61 (-946 to 696)	-0% (-5 to 4)	104
3	26,851 (25,400 to 28,507)	36,157	9,306 (7,650 to 10,757)	35% (27 to 42)	7,652
4	23,161 (22,042 to 24,677)	22,369	-792 (-2,308 to 327)	-3% (-9 to 1)	895
5	19,003 (18,064 to 20,126)	20,007	1,004 (-119 to 1,943)	5% (-1 to 11)	380
6	12,907 (12,140 to 13,884)	15,105	2,198 (1,221 to 2,965)	17% (9 to 24)	956
7	21,119 (19,777 to 22,709)	22,661	1,542 (-48 to 2,884)	7% (-0 to 15)	1,418

Table 5. Results of the sensitivity analysis correcting population for excess compared to the original analysis.

Output	Original analysis	Sensitivity analysis
Expected all-cause deaths	142,408 (95% CrI: 138,044 to 149,125)	141,464 (95% CrI: 136,750 to 148,572)
Excess all-cause deaths	13,786 (95% CrI: 7,068 to 18,149)	14,729 (95% CrI: 7,621 to 19,443)
Estimate of β_1	1.38 (95%CrI: 1.20 to 1.54)	1.38 (95%CrI: 1.22 to 1.54)
Estimate of β_2	0.96 (95%CrI: 0.92 to 1.00)	0.97 (95%CrI: 0.93 to 1.01)

S1.4 Separation model

We used a Bayesian hierarchical models to evaluate the association between all-cause deaths and laboratory-confirmed COVID-19-related deaths and expected all-cause mortality had the pandemic not occurred. Let O_{tk} be number all-cause deaths, L_{tk} the laboratory-confirmed COVID-19-related deaths and E_{tkq} the expected all-cause mortality had the pandemic not occurred, on the t-th week for the k-th group for the q-th sample (the group can be either one of the following: Total, age group, phase and cantons). Then the model is specified as follows:

$$\begin{aligned}
O_{tk} &\sim \text{Poisson}(\lambda_{tk}) \\
\lambda_{tk} &= \sum_k \beta_{1k} L_{tk} + \sum_k \beta_{2k} E_{tkq} + u_{tk} \\
u_{tk} &\sim \text{Normal}(0, \sigma_u^2), \text{ with } \sum_{tk} u_{tk} = 0 \\
\beta_{11}, \dots \beta_{1k} &\sim \text{Normal}(\beta_1, \sigma_1^2) \\
\beta_{21}, \dots \beta_{21} &\sim \text{Normal}(\beta_2, \sigma_2^2) \\
\beta_1 &\sim \text{Normal}(0^+, 2) \\
\beta_2 &\sim \text{Normal}(0^+, 5) \\
\sigma_1^2 &\sim \text{Exponential}(r_1 = 1) \\
\sigma_2^2 &\sim \text{Exponential}(r_1 = 0.1) \\
\sigma_u^2 &\sim \text{Exponential}(r_1 = 0.001)
\end{aligned} \tag{8}$$

$\beta_{11}, \dots \beta_{1k}$ and $\beta_{21}, \dots \beta_{2k}$ are the effects of laboratory-confirmed COVID-19-related deaths and expected all-cause mortality had the pandemic not occurred for the k-th subgroup. We used a multi-level structure on the betas, providing some level of smoothing by introducing a global mean. The global mean for $\beta_{11}, \dots \beta_{1k}$ is β_1 and comes from a truncated normal with 0 mean and 2 variance, whereas for for $\beta_{21}, \dots \beta_{2k}$ is β_2 and comes from a truncated normal with 0 mean and 5 variance. u_{tk} is an overdispersion term accounting for extra-Poisson variability and comes from a normal distribution with 0 mean and variance σ_u^2. Notice, that we impose sum-to-zero constraints for u_{tk} making sure the model has no intercept. All variance hyperparameters were assigned vague exponential priors with varying rates (r) depending on the scale of each random variable.

We repeat the above procedure for $q = 1, \dots, 200$, pooled the samples and took a random sample of 1000 realisations from the pooled sample.

Tables

Table S1: Median and 95% credible intervals of β_1 and β_2 for the Total group, the different phases and age groups considered.

	β_1			β_2		
	Median	2.5%	97.5%	Median	2.5%	97.5%
Total	1.38	1.20	1.54	0.96	0.92	1.00
Phase 1	1.46	1.02	1.90	0.94	0.89	0.99
Phase 2	2.00	0.22	7.65	0.97	0.92	1.03
Phase 3	1.46	1.28	1.64	0.93	0.84	0.99
Phase 4	0.42	0.01	1.71	0.93	0.86	0.99
Phase 5	2.41	0.80	4.92	0.98	0.92	1.06
Phase 6	2.31	1.37	3.28	0.98	0.90	1.08
Phase 7	1.64	0.60	2.91	0.95	0.86	1.03
Age group: 0-39	1.29	0.22	3.33	0.94	0.83	1.07
Age group: 0-59	1.11	0.11	1.80	0.88	0.81	0.95
Age group: 60-69	1.12	0.54	1.59	0.92	0.85	0.98
Age group: 70-79	1.20	0.98	1.42	1.01	0.95	1.06
Age group: $\geq$ 80	1.49	1.37	1.59	0.97	0.92	1.01

Table S2: Median and 95% credible intervals of β_1 and β_2 and total number of observed all-cause deaths across the different cantons.

		β_1			β_2			
Canton	Abbreviation	Median	2.5%	97.5%	Median	2.5%	97.5%	N
Aargau	AG	1.40	1.18	1.59	0.98	0.92	1.06	11489
Appenzell Innerrhoden	AI	1.45	1.14	1.89	0.95	0.84	1.06	310
Appenzell Ausserrhoden	AR	1.43	1.12	1.74	0.92	0.83	1.01	1097
Bern	BE	1.30	1.06	1.50	0.95	0.88	1.03	21333
Basel-Landschaft	BL	1.46	1.19	1.79	1.00	0.93	1.08	5717
Basel-Stadt	BS	1.48	1.24	1.83	0.91	0.81	0.99	4378
Fribourg	FR	1.40	1.21	1.59	0.96	0.89	1.05	5109
Geneve	GE	1.39	1.23	1.56	0.94	0.86	1.02	7901
Glarus	GL	1.43	1.10	1.77	0.94	0.84	1.03	900
Graubunden	GR	1.41	1.11	1.71	0.94	0.87	1.02	3877
Jura	JU	1.59	1.30	2.10	1.00	0.92	1.10	1715
Luzern	LU	1.46	1.23	1.78	0.98	0.92	1.08	7199
Neuchatel	NE	1.32	1.04	1.53	0.89	0.82	0.97	3591
Nidwalden	NW	1.46	1.14	1.83	0.97	0.87	1.05	760
Obwalden	OW	1.45	1.15	1.83	0.95	0.86	1.04	676
St. Gallen	SG	1.53	1.34	1.75	1.00	0.93	1.08	9844
Schaffhausen	SH	1.43	1.13	1.80	0.96	0.88	1.04	1787
Solothurn	SO	1.48	1.27	1.76	0.95	0.88	1.04	5426
Schwyz	SZ	1.50	1.25	1.81	0.99	0.92	1.08	2946
Thurgau	TG	1.35	1.12	1.53	0.94	0.88	1.01	4928
Ticino	TI	1.36	1.19	1.51	0.95	0.88	1.03	7627
Uri	UR	1.43	1.09	1.76	0.91	0.82	1.00	724
Vaud	VD	1.49	1.31	1.68	0.94	0.86	1.02	13057
Valais	VS	1.24	1.04	1.41	0.94	0.87	1.02	6507
Zug	ZG	1.56	1.29	2.01	0.97	0.89	1.04	1925
Zurich	ZH	1.38	1.14	1.61	0.97	0.90	1.04	25370

Table S3: Number of deaths by age group and phase of the pandemic.

	Observed deaths					
Phase	0-39	40-59	60-69	70-79	80+	Total
1	394	1254	1873	4008	13262	20791
2	422	1312	1876	3829	11664	19103
3	499	1817	3005	6862	23974	36157
4	437	1454	2296	4652	13530	22369
5	407	1403	2085	4128	11984	20007
6	257	915	1425	2994	9514	15105
7	377	1266	2012	4267	14739	22661
Total	2793	9421	14572	30740	98667	156193

Figures

Figure S1: Association between weekly laboratory-confirmed COVID-19-related deaths and absolute excess mortality by phase. The black line shows the slope of association corresponding to a 1 to 1 relation. The red lines show the association estimated with the model (corresponding to the β_1 coefficients, the full line represents the point estimate and the dashed lines the lower and upper bounds of the 95% credible interval).

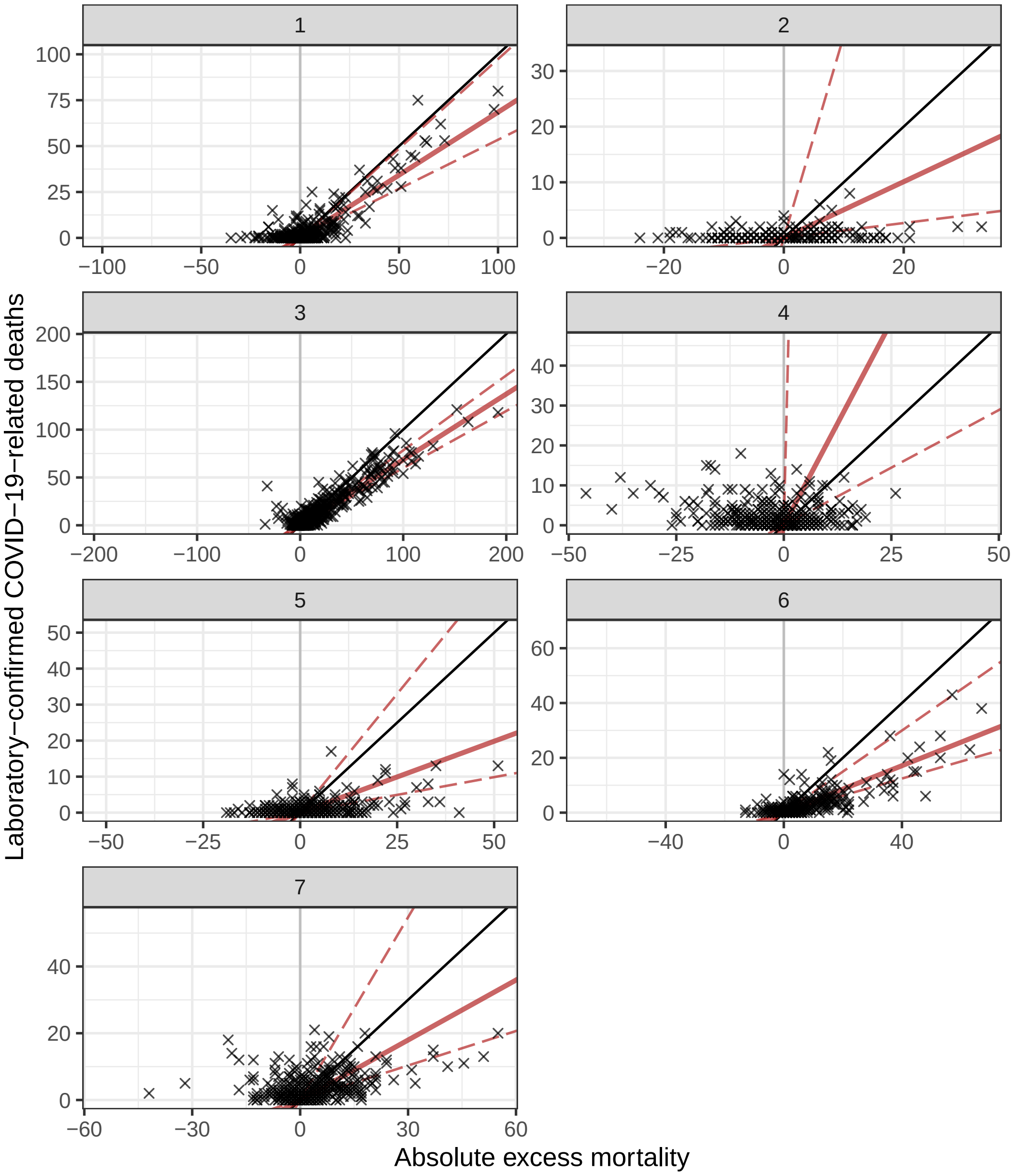

Figure S2: Mean and 95% credible intervals (error bars) of β_1 (panel A) and β_2 (panel B) across the different cantons. The canton abbreviations and sample size by canton are given in Table S2. n=156,193 number of observed all-cause deaths were used to derive the statistics.

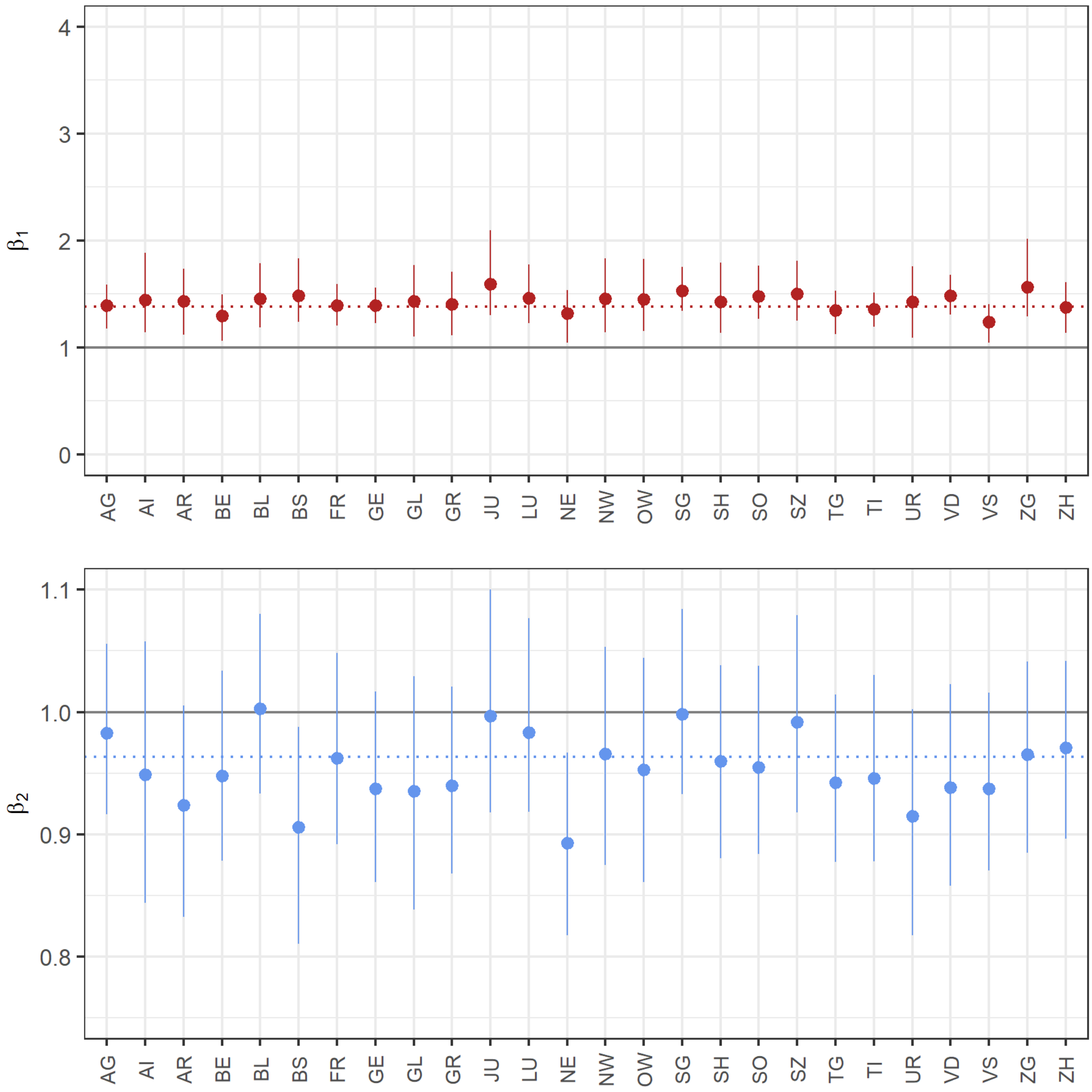

References

[1] Daniel Simpson, Håvard Rue, Andrea Riebler, Thiago G Martins, Sigrunn H Sørbye, et al. Penalising model component complexity: A principled, practical approach to constructing priors. *Statistical Science*, 32(1):1–28, 2017.

[2] Håvard Rue, Sara Martino, and Nicolas Chopin. Approximate Bayesian inference for latent Gaussian models by using integrated nested Laplace approximations. *Journal of the Royal Statistical Society: Series B (statistical methodology)*, 71(2):319–392, 2009.

[3] Garyfallos Konstantinoudis, Michela Cameletti, Virgilio Gómez-Rubio, Inmaculada León Gómez, Monica Pirani, Gianluca Baio, Amparo Larrauri, Julien Riou, Matthias Egger, Paolo Vineis, et al. Regional excess mortality during the 2020 covid-19 pandemic in five european countries. *Nature communications*, 13(1):1–11, 2022.

[4] Julian Besag, Jeremy York, and Annie Mollié. Bayesian image restoration, with two applications in spatial statistics. *Annals of the Institute of Statistical Mathematics*, 43(1):1–20, 1991.

[5] Andrea Riebler, Sigrunn H Sørbye, Daniel Simpson, and Håvard Rue. An intuitive Bayesian spatial model for disease mapping that accounts for scaling. *Statistical Methods in Medical Research*, 25(4):1145–1165, 2016.
